# Value-driven effects on perceptual averaging

**DOI:** 10.3758/s13414-022-02446-x

**Published:** 2022-02-09

**Authors:** Jaap Munneke, İlker Duymaz, Jennifer E. Corbett

**Affiliations:** 1 College of Health, Medicine, and Life Sciences, Brunel University London, Kingston Lane, London, UB8 3PH UK; 2 Centre for Cognitive Neuroscience, Brunel University London, London, UK; 3 Department of Psychology, Sabancı University, Istanbul, Turkey

**Keywords:** Perceptual averaging, Value-driven attention, Selection history

## Abstract

Perceptual averaging refers to a strategy of encoding the statistical properties of entire sets of objects rather than encoding individual object properties, potentially circumventing the visual system’s strict capacity limitations. Prior work has shown that such average representations of set properties, such as its mean size, can be modulated by top-down and bottom-up attention. However, it is unclear to what extent attentional biases through selection history, in the form of value-driven attentional capture, influences this type of summary statistical representation. To investigate, we conducted two experiments in which participants estimated the mean size of a set of heterogeneously sized circles while a previously rewarded color singleton was part of the set. In Experiment 1, all circles were gray, except either the smallest or the largest circle, which was presented in a color previously associated with a reward. When the largest circle in the set was associated with the highest value (as a proxy of selection history), we observed the largest biases, such that perceived mean size scaled linearly with the increasing value of the attended color singleton. In Experiment 2, we introduced a dual-task component in the form of an attentional search task to ensure that the observed bias of reward on perceptual averaging was not fully explained by focusing attention solely on the reward-signaling color singleton. Collectively, findings support the proposal that selection history, like bottom-up and top-down attention, influences perceptual averaging, and that this happens in a flexible manner proportional to the extent to which attention is captured.

## Introduction

The richness and complexity of the visual environment are such that the limited-capacity human visual system is incapable of in-depth processing all information available in a single glance. Rather, the visual system relies on heuristics and strategies to ensure that the world is perceived as stable and complete and that relevant information is selected and made available for further processing. To this end, the visual system relies on a host of complementary strategies, enabling it to circumvent these inherent capacity limitations. Two of these strategies referred to here are (1) perceptual averaging (also known as ‘ensemble encoding’ or ‘summary statistics’) and (2) visual selective attention. This study investigates how these qualitatively different mechanisms interact to help shape how we perceive the world around us.

Perceptual averaging refers to the ability of the visual system to rapidly extract higher-level statistics from sets of objects, such as the mean size or mean orientation of the set of objects (Ariely, 2001; Chong & Treisman, 2003, 2005a, b; Corbett & Oriet, 2011; Dakin & Watt, 1997; Oriet & Brand, 2013; Parkes et al., 2001). Rather than relying on in-depth processing of individual objects, perceptual averaging acts through encoding the statistical properties of the entire set of information as a whole (Alvarez, 2011; Ariely, 2001; Cohen et al., 2016), without processing the details of the individual elements. Perceptual averaging builds upon the observation that there is ample redundancy in the visual environment. Sets of similar objects are an integral part of the natural environment our visual system has to process at every waking moment: A flock of birds, a bowl of popcorn, or a bouquet of lilies are all composed of many individual but visually near-identical objects. The visual system can capitalize on this redundancy in the environment to circumvent processing capacity limitations (Alvarez, 2011).

Clear evidence for perceptual averaging was originally provided by Ariely (2001) who showed that when observers were shown a set of heterogeneously sized circles and asked to indicate whether a subsequently presented circle was present in the previous display of circles, they could not accurately distinguish member circles from nonmember circles. However, when observers were asked whether a newly presented circle was larger or smaller than the mean size of the previously shown set, they were surprisingly accurate. The main implications of this seminal study are that the visual system appears to be able to extract information about the set of objects without the need to acquire detailed information about the individual members of the set and that this process occurs in the absence of visual attention.

While perceptual averaging acts on a set of objects without processing the individual elements, visual selective attention takes the opposite approach and is used to selectively focus on a limited subset of the available visuo-sensory input for in-depth processing. Classic models of visual attention have distinguished between top-down and bottom-up mechanisms of attention, with top-down attention denoting the manner in which attention is voluntarily (i.e., goal-driven) focused on parts of the visual environment whereas bottom-up attention refers to a mechanism of attention that is automatic and driven by the perceptual qualities of a stimulus (i.e., stimulus-driven), such as its relative salience (for an overview of these mechanisms, see Corbetta & Shulman, 2002; Theeuwes, 2010). Recently, a third attentional mechanism, ‘selection history’, has been proposed in which attentional allocation is driven by lingering attentional biases towards stimuli that have been previously selected (Awh et al., 2012; Failing & Theeuwes, 2018; Theeuwes, 2019). Throughout the past decade, the intricate attentional properties of selection history have been predominantly scrutinized utilizing reward-based experiments in which attention is initially allocated to stimulus-features that signal reward, only to find that these features continue to capture attention even when reward is no longer available (also referred to as value-driven attentional capture; see, for example, Anderson, 2013; Anderson et al., 2011; Munneke et al., 2015, 2016). Despite these observations, questions remain as to the manner in which selection history biases our visual experience and how such a mechanism could interact with alternative information-processing strategies such as perceptual averaging.

Indeed, prior work has shown that visual selective attention can influence the manner in which perceptual averaging occurs. For example, a study by de Fockert and Marchant (2008) established that the size of an attended stimulus in a set can systematically bias the perceived mean size of that set in the direction of the size of the attended circle. In the first of two experiments, participants were shown sets of nine heterogeneously sized circles and asked, cued by a displayed prompt, to indicate on which side of the screen the largest or smallest circle of the set was presented, as well as which of the two subsequently presented circles represented the mean size of the set. By voluntarily guiding top-down attention to one side of the screen, the experiment tested the influence of top-down attention on perceptual averaging. In a second experiment, de Fockert and Marchant presented observers with sets of 12 gray circles, one of which was brighter than the others and therefore “popped out” from the display (i.e., a bottom-up feature singleton). Again, they asked participants to locate the pop-out circle and to indicate which one of two subsequent test circles was the correct representation of the mean size of the presented set of circles. By using pop-out targets, the experiment tested the influence of bottom-up attention on perceptual averaging. The results of both experiments demonstrated that when a set member was attended, either by top-down attention through cueing the size of the target, or by bottom-up attention exploiting the physical saliency of the target (i.e., brightness), mean size estimations became biased towards the size of the attended item. The study by de Fockert and Marchant, as well as other work (e.g., Chong & Treisman, 2005a) clearly shows a direct interaction between ‘classic’ modes of attentional selection and perceptual averaging.

Findings that top-down and bottom-up attention can affect mean size estimations strongly suggest that set representations might also be modulated by other qualities of stimuli that are prioritized by selective attention, such as selection history as indexed by value-driven attentional capture (Anderson et al., 2011). The influence of reward processing on perceptual averaging has recently been demonstrated in a study by Dodgson and Raymond (2020). In their study, participants first engaged in a reward-associated perceptual averaging task in which reward could be obtained as a function of color and performance with different colors signaling different rewards. In a subsequent perceptual averaging task, participants were shown a test array of 12 circles presented in three different colors (four circles per color), such that either the four largest, the four smallest, or the four medium-sized circles were presented in the high-reward color. Participants were asked to adjust a subsequently presented circle to the mean size of all 12 circles. Results clearly showed that the adjustment error (i.e., the difference between the set’s mean size and the participant-adjusted size) was biased by the subset of circles presented in the high-reward color. Logically, these findings were taken as evidence that indeed perceptual averaging is influenced by reward processing. However, despite reporting profound effects of reward processing on perceptual averaging, the precise attentional mechanism underlying these effects remain elusive. While value-driven attentional selection may play a role in the observed effects, the nature of attentional involvement is unclear at best. If value-driven attention is accountable for the observed effects, one has to question whether attention was allocated to a single high-reward element in the display, or to the entire set of high-reward circles. The latter seems unlikely as the used presentation duration (200 ms) is not of sufficient length to sequentially attend all four elements in the high-reward set. Alternatively, this bias may be completely attention-free and be fully driven by the mean size of the high-reward set (as opposed to attending its individual elements) using some form of subsampling from the display (see Myczek & Simons, 2008). As such, further investigation into the nature of reward-biased perceptual averaging is required.

The current study builds upon the work by Dodgson and Raymond (2020), by specifically investigating whether value-driven attentional capture can bias perceptual averaging. The precise aims of this study are twofold: (1) To investigate whether stimuli previously associated with reward influence perceptual averaging of a set of objects, such that high reward singletons bias the averaging process more than singletons with a lower value or no value. Such a finding would be in line with earlier work that shows that processing of a stimulus can be enhanced due to its association with a reward (e.g., Della Libera & Chelazzi, 2009). (2) To better understand the interplay between qualitatively different visuo-perceptual strategies that support the effective processing and interpretation of the overload of incoming visual information. Here, we conduct a study similar to the work by de Fockert and Marchant (2008), but rather than investigating top-down or bottom-up attention, we investigate whether selection history can influence perceptual averaging by adding a value-driven attentional component to the study, such that differently colored singletons are associated with different reward values. As such, rather than having an array of value-signaling stimuli that is potentially processed as a set (as in Dodgson & Raymond, 2020), there is only a single item (a color singleton circle) that signals reward, embedded in a set of heterogeneously sized circles. We expect the value-induced bias on perceptual averaging to scale with the value-level of the color singleton, with the largest bias for high-value color singletons and the smallest bias for no-value color singletons. In addition, we expect large stimuli to show a stronger bias on the perceived mean size, compared with small stimuli, purely due to the physical salience caused by the larger size (e.g., Treisman & Gormican, 1988). Given the observation that value-signaling stimuli can exert a stronger influence on attention than physical salience (i.e., bottom-up attention; Munneke et al., 2016), we further expect to find a difference between the influence of bottom-up attention as caused by the presence of a color singleton, and value-driven attention, such that the magnitude of the bias in perceptual averaging is larger for value-signaling color singletons as compared with singletons that do not signal value. Regardless, the application of bottom-up and value-driven attention to a set-member is expected to lead to a more biased average representation as compared with situations in which a salient or value-signaling stimulus is absent from the set.

## EXPERIMENT 1

### Methods

Participants conducted a perceptual averaging task that was designed to investigate the possible effects of the presence of reward-associated color singletons in a set of heterogeneously sized gray circles on the statistical mean size representations of the full set of circles (including the color singleton). The current study was aimed at investigating whether mean size representations were biased differentially by color singletons that were associated with different reward-levels (high reward, low reward, no reward).

#### Participants

There were 23 participants (four males) between 18 and 35 years of age, all of whom had normal or corrected-to-normal vision. All participants were recruited from Bilkent University’s undergraduate student population. All participants were compensated with eight Turkish Lira per hour for their participation and were able to earn a maximum of 11.5 Turkish Lira as reward according to their performance in the reward training task (see Training Task section for further details). All procedures were approved by Bilkent University’s ethical review board and were in line with the Declaration of Helsinki.

#### Apparatus

A Dell PC and a 21-in. NEC monitor (refresh rate: 60 Hz, resolution: 1,600 × 1,200 pixels) were used to display stimuli on a black background. Participants were positioned 57 cm from the screen, such that 1° of visual angle corresponded to 37 pixels. MATLAB (Version 2016a; The MathWorks, Natick, MA) and the Psychophysics Toolbox 3 (Brainard, 1997; Pelli, 1997) controlled all stimulus presentation and timing, response functions, and data collection.

#### Stimuli and procedure

**Fig. 1:**
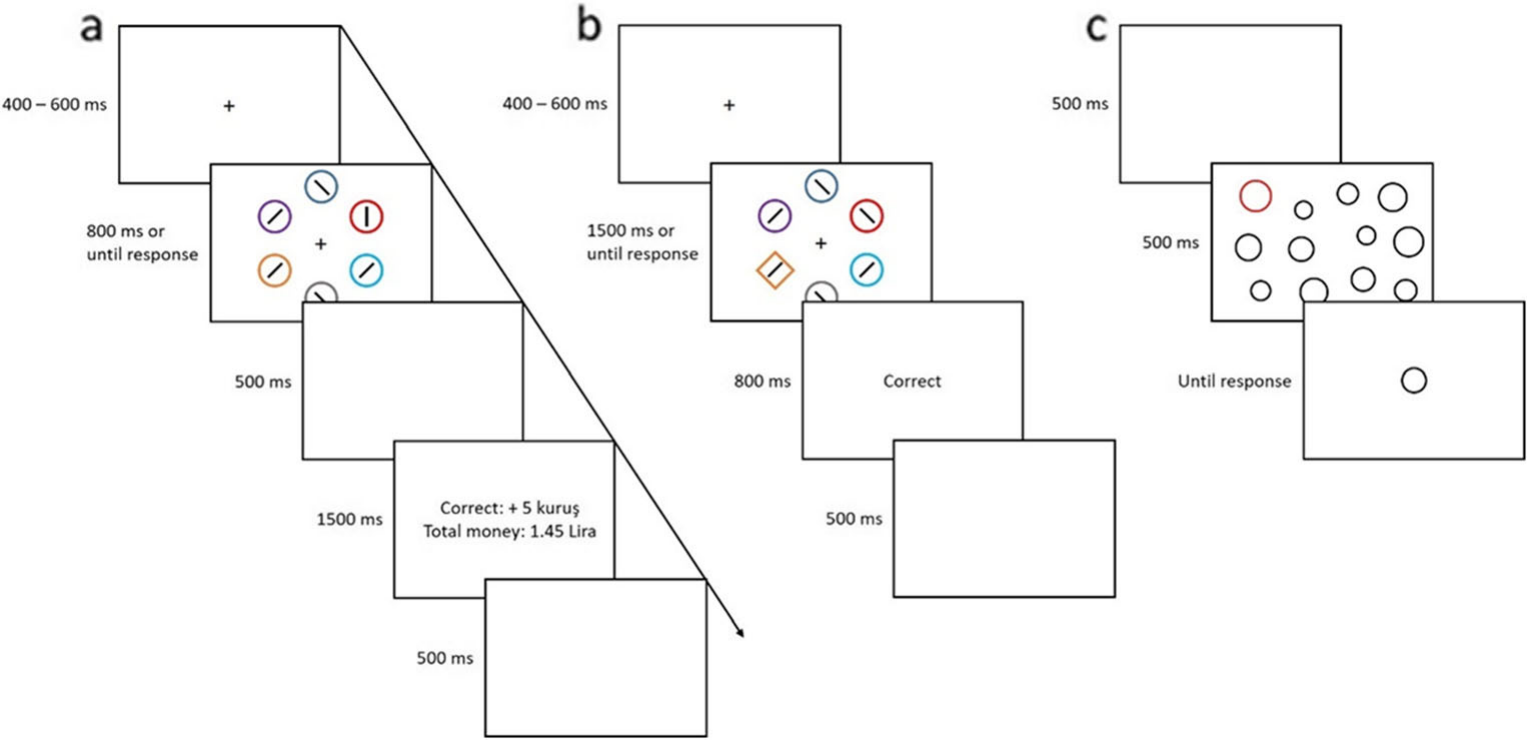
Time courses for (**a)** the training task, (**b)** the testing task, and (**c)** the perceptual averaging task

In this experiment, participants completed a series of three tasks. The training task (Fig. 1a) and testing task (Fig. 1b) were replications of Anderson and colleagues’ value-driven attentional capture paradigm (e.g., Anderson et al., 2011). These tasks were conducted to establish and test the association between a predefined reward level and a specific color. The third task was a perceptual averaging task (Fig. 1c) used to investigate the effect of value-driven attention on perceptual averaging. All three tasks were completed in a single session. The training task was always completed first, with the order of the testing and averaging tasks counterbalanced across participants. At the start of the training task, participants were informed that they could earn monetary rewards for fast and correct responses on each trial, the details of which are outlined below.

##### Training task (Fig. 1a)

The participants’ task was to report the orientation of a line centered in a red or green target circle among a set of five heterogeneously colored circles (Ø = 2.3°). The six circles were distributed equidistantly around a white fixation cross (0.2° × 0.2°) such that their locations coincided with an imaginary circle (Ø = 10°) on a black screen (approximating 0 cd/m^2^). On every trial, the five nontarget circles were each presented in a color chosen pseudorandomly without replacement from six possible colors (pink, blue, gray, brown, indigo, and yellow—equiluminant at 30 cd/m^2^). White lines (length = 0.6°, width = 0.1°) were presented in the center of each circle in different orientations. On each trial, the line inside the target circle was randomly drawn as either vertical (0°) or horizontal (90°), and the lines inside the nontarget circles were diagonals, each randomly tilted by ±45°. Participants were asked to report the orientation of the line inside the target circle by pressing either the ‘z’ key for a vertical line or the ‘m’ key for a horizontal line. Red and green circles acted as high reward and low reward targets, with reward-color combinations counterbalanced over participants. Red and green target circles were presented with equal probability (50%). On 80% of the high-reward trials, participants could earn a monetary reward of 5 Kuruş (0.05 Turkish Lira) if they correctly reported the orientation of the line inside the target within 800 ms after the target onset. In the other 20% of the high-reward trials, they could earn 0.5 Kuruş (0.005 Turkish Lira) for prompt and correct responses. On trials with a target that signaled low reward, these proportions were reversed such that participants earned 5 Kuruş for fast and correct responses on 20% of low-reward trials and 0.5 Kuruş on 80% of low-reward trials.

The task consisted of eight blocks of 60 trials each, the first block being a practice block. Each trial began with a fixation cross displayed for a random duration of 400, 500, or 600 ms. Subsequently, the search display was presented either until the participant responded, or until 800 ms had passed. Responses made after 800 ms were recorded as incorrect answers. After each trial, participants were presented with written feedback about their accuracy (Correct or Incorrect), the amount of monetary reward obtained if the answer was correct and in time, and the cumulative amount of money earned so far throughout the experiment. Feedback was displayed in the center of the screen for 1,500 ms, followed by a blank screen displayed for 500 ms, after which the next trial automatically started. At the end of each block, feedback (total amount of money earned, total block accuracy) and instructions to rest for at least 10 seconds before beginning the next block were displayed in white text on a black background. After the mandatory resting duration, participants were able to initiate the next block by pressing the spacebar. Before beginning the training task, participants were informed that they had to complete the task with an overall accuracy of at least 75% to be able to continue to the subsequent testing and averaging tasks. This cut-off criterion was established to ensure that robust color–reward associations would be formed.

##### Testing task (Fig. 1b)

On each trial in the reward testing task, participants were instructed to report the orientation of a line inside the unique shape in a radial display of six shapes. Displays consisted of radial arrays of either one circle (Ø = 2.3°) and five diamonds (one side = 2.3°) or one diamond and five circles distributed equidistantly around a white fixation cross (0.2° × 0.2°), such that their locations coincided with an imaginary circle (Ø = 10°) on a black screen. The unique target shape contained either a horizontal or a vertical line, whereas nontarget shapes contained ±45°diagonal lines similar to the training task. Participants pressed the ‘z’ on the keyboard to report a vertical line, and the ‘m’ key for a horizontal line. On a random half of the trials in each block, one of the nontarget shapes was presented in one of the colors previously associated with high or low reward (red or green). The other half of the trials had no value-associated distractors. Nondistractor shapes and targets had unique colors chosen pseudorandomly from the same six colors that were used in the training task. Participants were clearly informed that no additional reward could be obtained in the testing task.

The testing task consisted of two blocks of 120 trials each, preceded by a practice block of 120 trials. Each trial began with a fixation cross displayed for a random duration of 400, 500, or 600 ms, followed by the presentation of the stimulus display, which lasted for 1500 ms, or until participant input. After every trial, participants were presented with feedback about their trial accuracy (Correct or Incorrect). The feedback screen was displayed for 800 ms, after which the next trial was automatically initiated following a 500 ms blank screen interval. At the end of every block, participants were instructed to rest for at least 10 seconds. After the mandatory 10-second break, they could press the spacebar to continue with the next block.

##### Perceptual averaging task (Fig. 1c)

On each trial in the perceptual averaging task, participants were briefly presented with a set of 12 heterogeneously sized circles and subsequently asked to estimate the mean size of the set (i.e., what was the mean size of the 12 circles in the previous screen). A random 75% of trials had 11 gray circles and one colored circle. One-third of the trials with a colored circle contained a circle presented in a color previously associated with a high reward, one-third had a circle presented in a color previously associated with a low reward, and one-third had a circle presented in a novel, nonrewarded color. In the trials with color singleton circles, the colored circle could be either the largest or smallest circle in the set. The final 25% of trials contained 12 gray circles and no colored circle was presented on these trials. Across all trials, the 12 circles were equiluminant.

Each circle in the display was presented within an imaginary 4 × 4 square array, subtending 20° × 20° of visual angle. The positions of the individual circles were jittered on each trial by a random value from −0.75° to 0.75^o^, in 0.05° steps in both the horizontal and vertical direction, with the restriction that individual circles could not overlap. On each trial, circle diameters were drawn without replacement from a base array ranging from 0.7° to 2.9° in 0.2° steps. The resultant array of trial values was multiplied by a random constant between 0.7 and 1.3, to ensure that participants were not repeatedly presented with the same mean size over successive trials.

Each display of 12 circles was presented for 500 ms, followed by an adjustable test circle (frame width = 0.125°). On each trial, the initial size of the test circle was pseudorandomly selected such that the starting size fell between the smallest and the largest circle size for the given trial. Participants used the left and right arrow keys to adjust the size of the test circle (right arrow to increase size, left arrow to decrease size), and confirmed their final answer by pressing the Enter key. This task focused on accuracy and was nonspeeded. The experiment automatically proceeded to the next trial after a 500-ms blank display following the participant’s response. To ensure participants were actively engaged in the task, they received a warning in red text if they pressed the Enter key without adjusting the circle properly (i.e., they made fewer than three arrow-key presses). Participants completed five blocks of 40 trials each in the averaging task. The first block was treated as a practice block. As in the training and testing tasks, participants were obliged to take a 10-second break between blocks, and then were able to continue to the next block by pressing the spacebar. In addition, they were instructed to carefully adjust the test circle on each trial, and that failure to adjust the circle appropriately would lead to expulsion from the experiments (after five red screens). No participants were dismissed for failing to properly adjust the test circle in the averaging task.

Before beginning each of the three tasks, participants were presented with written, illustrated instructions in their language of choice (Turkish or English), and the experimenter ensured they fully understood each of the tasks before they were allowed to proceed to the main experimental blocks. In the training and testing tasks, the experimenter and the written instructions stressed that participants should respond as quickly and accurately as possible. In the perceptual averaging task, participants were instructed to respond as accurately as possible. Data from all practice blocks were excluded from any further analyses.

### Results

Out of the 23 participants, one failed to reach the mandatory 75% accuracy threshold in the training task and one participant withdrew from the experiment following the training phase. Data from these two participants have been removed from all analyses. One participant did not finish the perceptual averaging task and was excluded from all data analyses as well. Presented results include only the data from the remaining 20 participants.

#### Training task

**Fig. 2:**
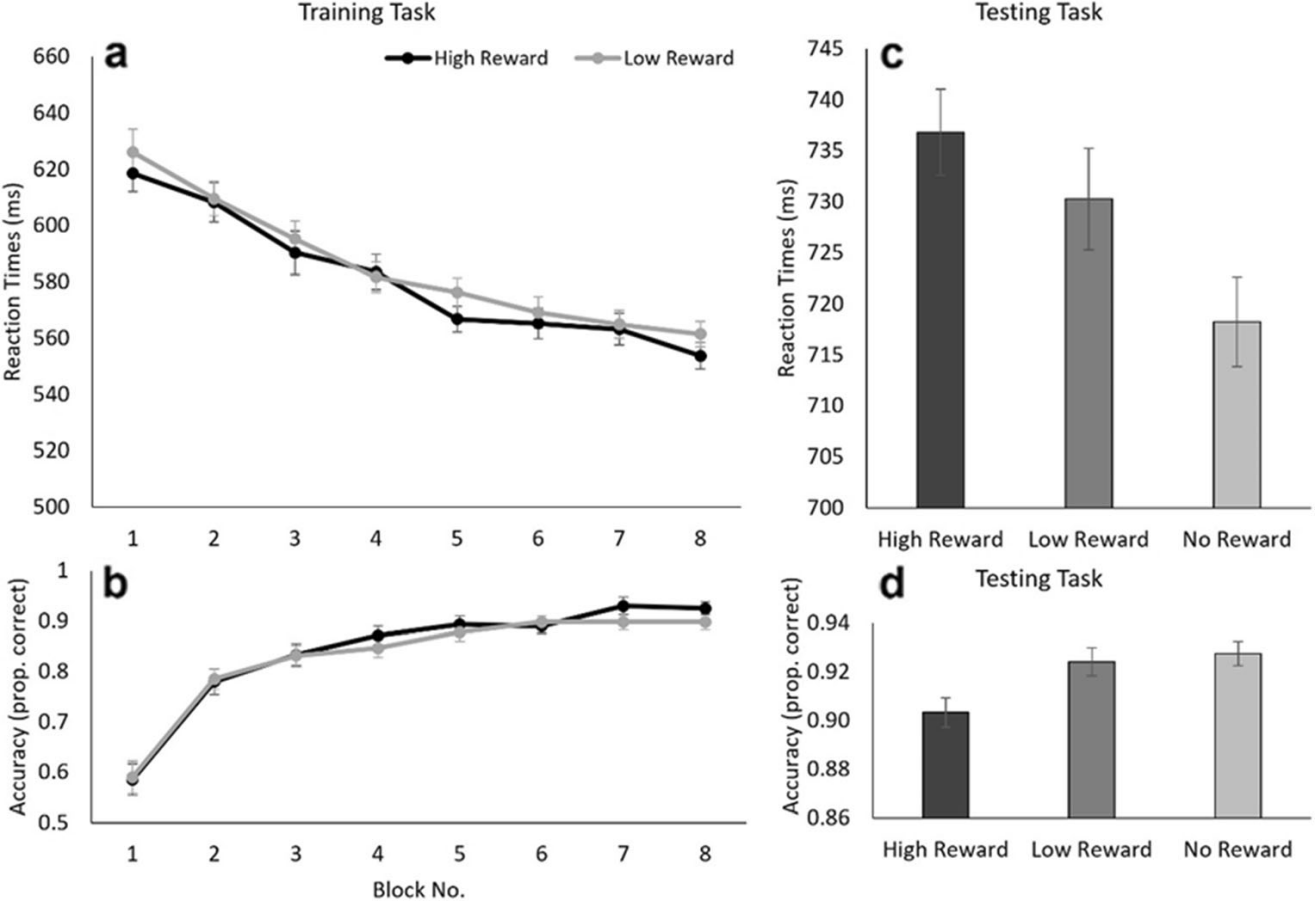
Experiment 1. Average reaction times and accuracy scores) for the reward training (**a** and **b**) and testing task (**c** and **d**). Error bars indicate 95% confidence intervals corrected for the use of within-subject designs (Cousineau, 2005; Morey, 2008).

To confirm whether the training task was efficient in associating target colors with reward, a repeated-measures ANOVA with Reward Level (high reward, low reward) and Block Number (2–8) as factors was conducted on the reaction times from the training task. Only trials in which the participants responded accurately (13.14% of the trials removed) with reaction times greater than 200 ms and within three standard deviations of the participant’s conditional mean (high reward, low reward) were included in all analyses (0.08% of the trials removed). Results of the ANOVA showed a main effect of Block Number, *F*(6, 114) = 20.158, *p* < .001, η_p_^2^ = .515, indicating that participants were able to find and respond to the targets faster as they progressed through the experimental blocks (see Fig. 2a); a finding which was supported by a significant linear trend of Block Number, *F*(1, 19) = 73.350, *p* < .001, η_p_^2^ = .794. There was neither a main effect of Reward Level (*F* < 1), nor a significant interaction between the two factors (*F* < 1).

Similar results were obtained for the non-truncated accuracy scores, demonstrating only a main effect of Block Number, *F*(6, 114) = 14.694, *p* < .001, η_p_^2^ = .436, such that participants became more accurate at correctly identifying the target as they progressed through the experiment (see Fig. 2b). This is supported by a significant linear trend over Block Number, *F*(1, 19) = 30.250, *p* < .001, η_p_^2^ = .614. Neither a main effect of Reward Level, nor an interaction between the two factors was observed (both *F*s < 1).

#### Testing task

The testing task was conducted to investigate whether the stimulus-reward contingencies influenced attentional allocation in the absence of reward (similar to Anderson et al., 2011). A repeated-measures ANOVA was conducted on the reaction times from the testing task with Distractor Type (high reward, low reward, and no reward) as the within-subjects factor. Only trials in which the participant responded accurately (7.94% of the data discarded) with reaction times greater than 200 ms and within three standard deviations of the participant’s conditional mean (high reward, low reward, and no reward) were included in all analyses (0.93% of the data discarded). Reaction times to the target differed between the three distractor conditions (High Reward: 737 ms, Low Reward: 730 ms, No Reward: 718 ms), as evidenced by a significant effect of the Distractor Type, *F*(2, 38) = 3.501, *p* = .040, η_p_^2^ = .156. Uncorrected planned-comparisons confirmed that the presence of a high reward distractor significantly slowed reaction times compared with trials that contained no reward-associated distractors, *t*(19) = 2.932, *p* = .009. Reaction times for the low reward distractor condition did not differ significantly from those of the no-reward condition, *t*(19) = 1.581, *p* = .130, nor from the high-reward condition, *t*(19) = 0.890, *p* = .384 (see Fig. 2c).

A similar analysis on the accuracy scores revealed a main effect of Distractor Type, *F*(2, 38) = 4.374, *p* = .020, η_p_^2^ = .187, with uncorrected planned *t* tests showing that participants were less accurate in the high-reward condition (prop. correct = .903) as compared with the low-reward condition (prop. correct = . 924), *t*(19) = 2.123, *p* = .047, as well as a significant different results between the high-reward and the no-reward condition (prop. correct = 0.927), *t*(19) = 2.820, *p* = .011 (Fig. 2d). No significant difference between the low reward and no reward condition was observed, *t*(19) = 0.412, *p* = .685.

#### Perceptual averaging task

To examine whether a value-associated color singleton influenced participants’ abilities to accurately estimate the mean size of a set of circles, we conducted a repeated-measures ANOVA on the absolute mean error scores calculated separately for each participant and condition.^1^ Error scores were defined as the absolute difference between the user-adjusted estimated mean size, and the actual mean size including the color singleton. Note that we have not presented error directionally because this approach averages out the effects of interest due to the well-established finding that observers overestimate mean size (e.g., Choi & Chong, 2020), with the only known exception reported for individuals diagnosed with autism spectrum disorder (Corbett et al., 2016). No additional data trimming was conducted, and no error data were removed from the dataset.

**Fig. 3:**
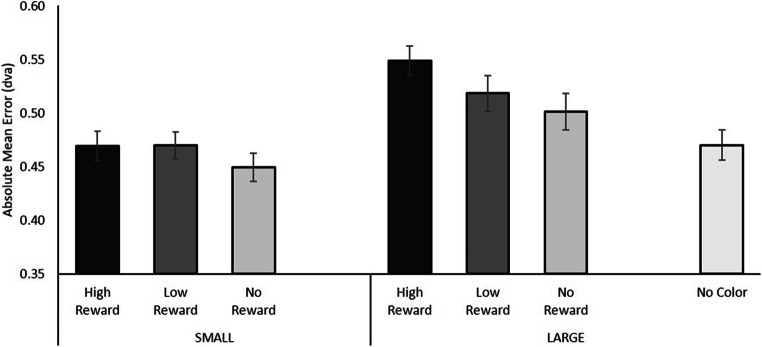
Results of the Averaging Task in Experiment 1. The average of the participant’s absolute mean error values was larger for large circles compared with small circles and scaled with reward level. The rightmost bar indicates the average error when no color singleton was present. Error bars indicate 95% confidence intervals corrected for the use of within-subject designs (Cousineau, 2005; Morey, 2008)

A repeated-measures ANOVA was conducted using Reward Level (high reward, low reward, no reward—the no-distractor condition was left out of this analysis) as indicated by the color of the singleton present in the display and Singleton Size (large, small) as factors. A main effect of Singleton Size was observed, *F*(1, 19) = 17.467, *p* = .001, η_p_^2^ = .479, indicating that the overall error was larger for trials that contained a large color singleton (error = 0.523°), compared with trials in which a small color singleton was present (error = 0.463°). A significant main effect of Reward Level was observed, *F*(1, 19) = 2.524, *p* = .046, η_p_^2^ = .117 (one-tailed^*^^2^), suggesting that value-driven attentional capture has a significant influence on perceptual averaging (Fig. 3). This main effect of Reward Level is further substantiated by a linear trend of increasing accuracy with decreasing reward magnitude, *F*(1, 19) = 5.232, *p* = .034, η_p_^2^ = .216. No interaction between Singleton Size and Reward Level was observed, *F*(1, 19) = 1.139, *p* = .331, η_p_^2^ = .057. The lack of an interaction effect between Singleton Size and Reward Level was further corroborated by Bayesian testing, modelling the fit of the data when including only the main effects of both Singleton Size and Reward Magnitude, with the model that adds the interaction of those two terms as well. Compared with H_0_, results showed a better fit for the model containing only the two main effects (BF_10_ = 5142.104) compared with a model that includes the interaction term as well (BF_10_ = 1172.097), such that the model without the interaction term is 4.39 times more likely to explain the data as compared the model that includes the interaction term.

Two sets of uncorrected planned comparisons were performed. First, paired-samples *t* tests between each of the color–reward levels and the no-singleton condition (i.e., no color singleton was present in the display) revealed that the presence of a large high-reward or a large low-reward color singleton yielded significantly larger errors compared with when no colored singleton was present in the display (no-singleton: 0.47°, large high reward: error = 0.55°), *t*(19) = 4.952, *p* < .001; (large low reward: error = 0.52°), *t*(19) = 2.110, *p* = .048. Interestingly, the large no reward color singleton (error = 0.50°) did not yield a significant difference, compared with when no colored singleton was present in the display, *t*(19) = 1.257, *p* = 0.224. No significant differences between any of the other colored singletons and the no-singleton condition were observed (smallest *p* = .298). Second, paired-samples *t* tests were conducted between the different reward magnitudes of equally sized color singletons, further testing the influence of reward on averaging, independent of singelton size (i.e., separately for large and small color singletons). These tests yielded only one significant result, indicating a difference in error between the large high-reward and the large no-reward condition (0.55° vs. 0.50°), *t*(19) = 2.341, *p* = .030. None of the other comparisons among large or small singletons yielded significant results (smallest *p* = .192).

### Discussion

Experiment 1 yielded two main observations: (1) The estimation of the mean size of the set of circles is strongly modulated by the size of the attended stimulus (similar to de Fockert & Marchant, 2008), with large color singletons producing larger errors than small color singletons. (2) Results show that perceptual averaging of a set of heterogeneously sized circles can be influenced by stimuli that signal reward, such that the averaging process is biased proportional to the reward magnitude. This is best observable for the use of large color singletons where both a high and low reward singleton yielded larger errors compared with the condition in which no color singleton was presented whereas no such effect was observed for color singletons that did not signal a reward. Additionally, the large high-reward singleton yielded larger errors compared with the large no-reward singleton, indeed suggesting that reward influences the averaging process.

Interestingly, when a large color singleton was presented that was not associated with reward, the observed error was not significantly different from the no-color condition; an observation that seems to be inconsistent with findings observed by de Fockert and Marchant (2008). An explanation as to why the current results differ from those observed by de Fockert and Marchant may be related to the nature of the used tasks and the manner in which attention is manipulated. A number of key differences between the current task and the task by de Fockert and Marchant stand out: (1) The brightness singleton in the task by de Fockert and Marchant acts as a target, whereas the color singleton in the current study has no intrinsic function to the task at hand. That is, participants in the study by de Fockert and Marchant are asked to select this pop-out target and respond to its location on the screen. In the current study, no such selection needs to take place to conduct the task, which potentially could diminish the singleton’s influence on the averaging process. Alternatively, presenting color singletons that previously signaled reward may activate a stronger response on a priority map (i.e., bottom-up + selection history) such that these particular stimuli modulate the averaging processes similar to the target pop-out stimuli used in de Fockert and Marchant’s study. (2) The bottom-up quality of the current color singletons can be debated. The colors of the different singletons are always equiluminant with the surrounding gray circles such that only chromaticity, but not saliency acts as a bottom-up feature. While chromaticity alone can capture attention (e.g., Turatto & Galfano, 2001), luminance arguably has a stronger effect on attentional capture (e.g., Irwin et al., 2000). As such, a color-singleton that is otherwise equally salient may not capture attention effectively unless associated with a secondary attention capturing quality such as its association with reward. (3) The set of circles in the study by de Fockert and Marchant were presented for 1,000 ms, whereas in the current experiment they are presented for only 500 ms. However, while longer exposure to the set of circles provides more time to encode the individual elements of a set, it is unlikely to have an effect on the average set representations as Whiting and Oriet (2011) show that averaging performance does not increase after 200 ms of exposure. Nevertheless, the reduced presentation duration could have diminished the influence of the nonrewarded color singleton on the averaging process. (4) Participants in the experiment by de Fockert and Marchant were expected to conduct a dual-task where they first had to engage in a cued search task finding either the largest or the smallest circle in the display and responding to its location on the screen (left or right). Subsequently, participants had to select one of two newly presented circles and indicate which of the two circles represented the mean of the set of circles. This dual-task set-up is qualitatively different from the current work in that the attended small or large circle in the experiment by de Fockert and Marchant is task relevant unlike the current experiment where the color singleton is part of the set and does not need to be attended. The lack of task relevance, combined with the reduced saliency of the color singleton in the current study may well be the reason that non-rewarded color singletons do not observably bias the perception of the mean size of the set.

Importantly, we did not observe a significant difference between the influence of high and low reward-associated color singletons on perceptual averaging. While not all value-driven attentional capture studies show a lingering difference in attentional bias between high and low value-signaling stimuli (e.g., Anderson & Yantis, 2012; Bucker & Theeuwes, 2017), when such differences are observed, they are often taken as evidence that value, rather than some other bottom-up or top-down factor is the driving force underlying value-driven capture (but see Munneke et al., 2020, for an alternative interpretation). In Experiment 1, one of the reasons why no difference in absolute error between the high- and low-reward conditions is observed could be evoked by the nature of the experiment. Prior work has hypothesized that the main performance difference between high- and low-reward conditions in value-driven capture experiments is potentially attributed to the amount of attentional resources allocated to the different value-signaling color singletons, with more resources allocated to high- compared with low-reward stimuli. However, when such value-driven performance differences are observed, the attention capturing stimuli often act as task irrelevant distractors that are attended while searching for a target stimulus. In Experiment 1, there is no target stimulus to be found and its absence allows participants to attend the color singleton and stay focused at this location for the remainder of the trial, with differences in attentional demands negated due to not being engaged in a different, attention demanding task. To test this hypothesis, in Experiment 2, we included a secondary task in addition to the perceptual averaging task. A small line segment was presented inside one of the circles in the display and participants had to respond to its orientation, forcing participants to allocate attention to the nonrewarded stimuli in search for a target stimulus.

## EXPERIMENT 2

### Methods

#### Training and testing task

The tasks used to establish and test color–reward associations were similar to those presented in Experiment 1, with one notable difference. In Experiment 1, only red and green stimuli were used as potential rewarded target colors in the training task and as such were presented as distractors in the testing task, where a third color was added (blue) to investigate the influence of physical saliency in the absence of reward. As all colors were equiluminant, the color distribution within the experiment should not systematically influence results. However, to further guard against any differences that may have been specific to the three colors, the reward associated with the three colors (red, green, blue) was counterbalanced over participants in Experiment 2, such that each color was equally often associated with high, low and no reward.

#### Perceptual averaging task

The averaging task was the same as in Experiment 1, with one notable exception. On each trial in Experiment 2, one of the 12 circles contained either a horizontal or vertical line segment (0.06° × 0.35°). The line segment was never presented in the two smallest or two largest circles and therefore never coincided with the color singleton (which was always the largest or the smallest circle in the set). Participants were asked to find this target stimulus and discriminate its orientation, but to withhold this response until after they had adjusted the subsequently presented test circle (similar to Experiment 1). Once participants had finished adjusting the test circle size, a new screen appeared, prompting them to respond whether the presented line segment was horizontal (press ‘x’) or vertical (press ‘z’). Responses to the averaging section of the experiment were made using the right hand, whereas responses to the line-orientation task were made using the left hand. Due to the nature of the experiment, the response to the line-segment’s orientation was unspeeded, and only accuracy was measured.

### Results

Out of 24 participants, three failed to reach the mandatory 75% accuracy threshold in the training task, and two experienced technical problems during the experiment, resulting in their data being removed from all further analyses. All analyses were conducted using the data from the remaining 19 participants.

#### Training task

**Fig. 4:**
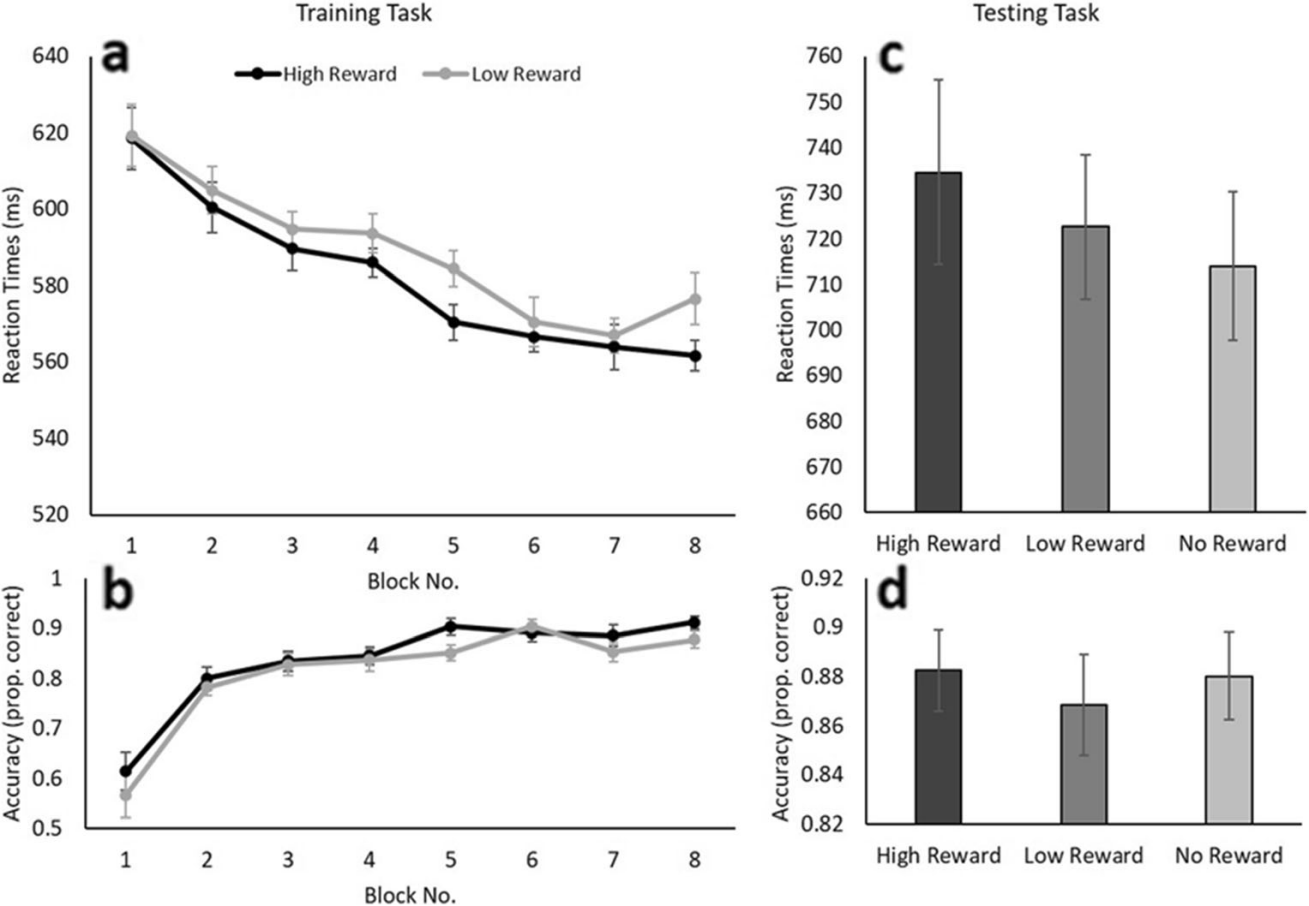
Average reaction times (top) and accuracy scores (bottom) over block number for the reward training task in Experiment 2. Error bars indicate 95% confidence intervals corrected for the use of within-subject designs (Cousineau, 2005; Morey, 2008)

Similar to Experiment 1, a repeated-measures ANOVA with Reward Level (high reward, low reward) and Block Number (2–8) as factors was conducted to confirm whether the training task was efficient in associating target colors with differential reward levels. Only trials in which the participants responded accurately (14.25% of the trials removed) with reaction times greater than 200 ms and within three standard deviations of the participant’s conditional mean (high reward, low reward) were included in all analyses (0.13% of the trials removed). Results of the ANOVA on the reaction time data indicated a main effect of Block Number, *F*(6,108) = 15.999, *p* < .001, η_p_^2^ = .471, once again showing that participants started responding faster while working through the different blocks of the experiment (see Fig. 4a). The decrease in reaction times over experimental blocks was further supported by a significant linear trend, *F*(1, 18) = 58.369, *p* < .001, η_p_^2^ = .764. A main effect of Reward Level was observed, *F*(6, 108) = 3.524, *p* < .038, η_p_^2^ = .164 (one-tailed), indicating that participants responded slightly faster to targets associated with a high reward (577 ms) compared with targets associated with a low reward (585 ms). No interaction between the two factors was observed (*F* < 1). A similar ANOVA on the accuracy data yielded only a main effect of Block, *F*(6, 108) = 11.218, *p* < .001, η_p_^2^ = .384, suggesting that participants became more accurate in responding to the target while going through the different blocks of the Experiment (see Fig. 4b); a result which was supported by the observation of a significant linear trend of Block, *F*(1, 18) = 43.860, *p* < .001, η_p_^2^ = .709. No differences in accuracy were observed for the different Reward Levels, *F*(6, 106) = 1.658, *p* = .214, η_p_^2^ = .084, nor did the interaction between the two factors reach significance, *F*(6, 108) = 1.154, *p* = .336, η_p_^2^ = .060.

#### Testing task

To confirm that color–reward associations could influence attentional allocation in the absence of reward, a repeated-measures ANOVA was conducted on the obtained reaction times, with Distractor Type (high reward, low reward, no reward) as the only factor. Prior to the analyses, incorrect trials (12.21%) and trials with response times below 200 ms or three standard deviations above the participant’s conditional mean (0.87%) were removed from the dataset. The ANOVA yielded a main effect of Reward Level, *F*(2, 36) = 3.665, *p* = .036, η_p_^2^ = .169, with uncorrected planned t-tests showing that the presence of a color singleton circle in the previously high rewarded color (735 ms) led to significantly slower reaction times as compared with trials in which a color not associated with reward (714 ms) was used, *t*(18) = 2.373, *p* = .029. While differences between High and Low Reward (723 ms) *t*(18) = 1.622, *p* = .122., and Low and No Reward, *t*(18) = 1.282, *p* = .216. were not significant, a linear trend over Reward Level was observed, showing increasingly faster reaction times with diminishing reward levels, *F*(1, 18) = 5.631, *p* = .029, η_p_^2^ = .238 (see Fig. 4c). No effect of Reward Level on the accuracy scores was observed in a follow-up repeated-measures ANOVA (*F* < 1; see Fig. 4d).

#### Perceptual averaging task

**Fig. 5:**
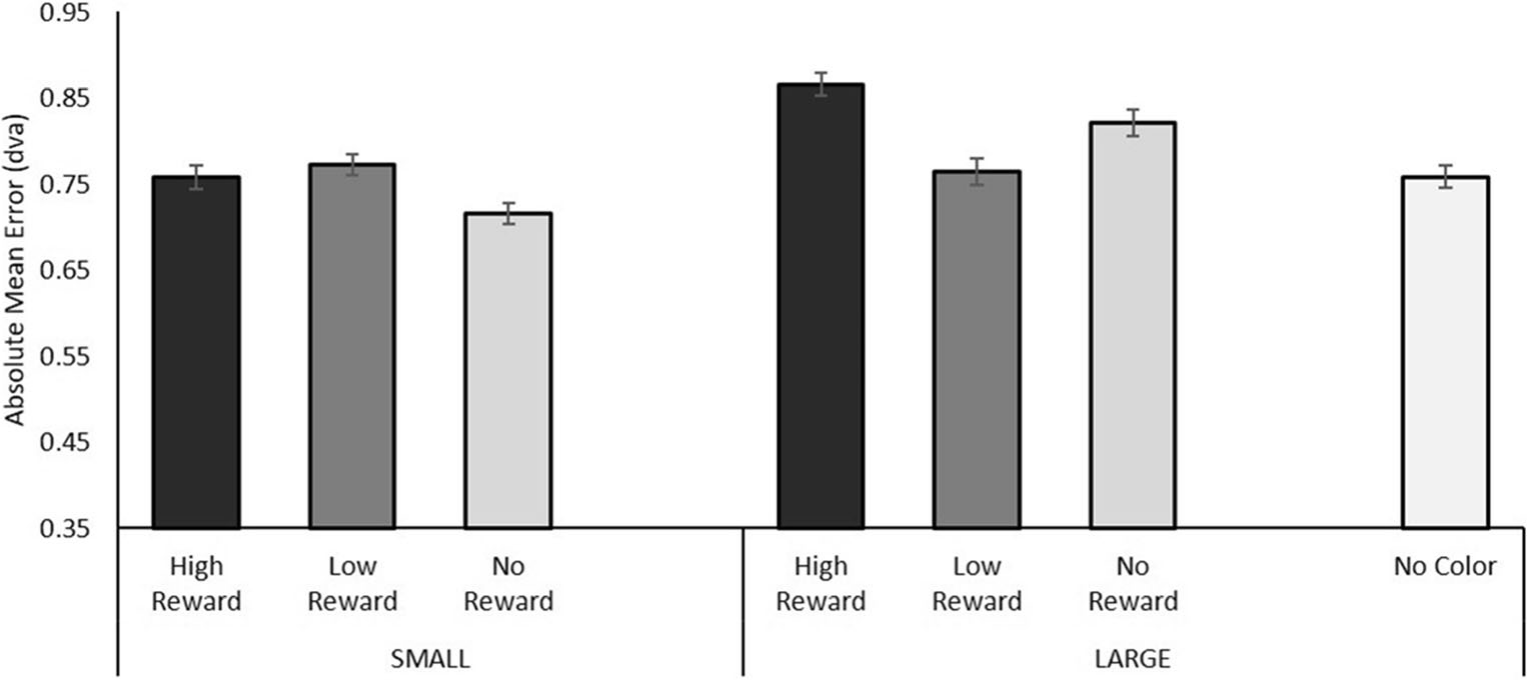
Results of the Averaging Task in Experiment 2. The average absolute error was larger for large circles compared with small circles, in particular for large circles associated with a high reward. Error bars indicate 95% confidence intervals corrected for the use of within-subject designs (Cousineau, 2005; Morey, 2008)

To investigate whether the effects of a reward-associated color singleton on the perception of the mean size of a set of circles was influenced by the necessity to disengage from the capturing value-associated singleton, a repeated-measures ANOVA on the absolute mean error values was conducted.^3^ Values were calculated in an identical fashion to Experiment 1, and data were nontruncated. A repeated-measures ANOVA with Reward Level (high reward, low reward, no reward—we excluded the no-color singleton condition here) and Singleton Size (large, small) as factors was conducted. This analysis included only those trials in which participants correctly identified the orientation of the line segment in the secondary task (average accuracy: 90.2%; 9.8% of the data discarded). Similar to Experiment 1, a significant difference in absolute mean error was observed for the different sizes of the color singleton, *F*(1, 18) = 6.021, *p* = .025, η_p_^2^ = .251, such that the overall error was larger for trials that contained a large color singleton (error = .82°) compared with trials with a small color singleton (error = .75°). No significant main effect of Reward Level was observed, *F*(2, 36) = 1.994, *p* = .151, η_p_^2^ = .100, nor did the interaction yield significant results, *F*(2, 36) = 2.338, *p* = .111, η_p_^2^ = .115 (Fig. 5). The lack of an interaction effect between Singleton Size and Reward Level was again further analyzed using Bayesian testing, modelling the fit of the data when including only the main effects of both Singleton Size and Reward Magnitude and comparing that with the model that adds the interaction of those two terms as well. Compared with H_0_, results showed a better fit for a model containing only the two main effects (BF_10_ = 7.634) compared with a model that includes the interaction term as well (BF_10_ = 3.724), such that the model without the interaction term is 2.05 times more likely to explain the data as compared the model that includes the interaction term.

Similar to Experiment 1, two sets of uncorrected planned comparisons were conducted. We chose to include these comparisons despite not finding a significant interaction between Singleton Size and Reward Level, as we expected that subtle effects of reward could be observed within the different large-circle conditions; effects that could be absent for the smaller singleton sizes, without this potentially showing up in any interaction. Indeed, prior work has shown that larger feature values attract attention more efficiently among smaller feature values than vice versa (Treisman & Gormican, 1988). Regardless, do note that these comparisons do not follow directly from a significant interaction and the results should be interpreted tentatively. First, using paired-samples *t* tests different reward levels for equally sized color singletons were compared. This analysis showed that the presence of large high-reward singletons yielded larger errors than large low-reward singletons, *t*(18) = 2.453, *p* = .025. None of the other comparisons yielded significant results (small circles, smallest *p* = .095; large circles, smallest *p* = .203). Second, further evidence that the presence of a large high-reward circle resulted in the largest absolute error was given by a series of additional planned comparisons in which the mean accuracy for each singleton type (separately for singleton size and reward level) was compared with the no-singleton condition. The large high-reward circle showed a mean absolute error that was significantly different from the no-color singleton baseline, *t*(18) = 2.875, *p* = .010, but none of the other comparisons against baseline showed significant differences (small circles: smallest *p* = .126; large circles: smallest *p* = .088).

### Discussion

The results of Experiment 2 partially replicated those of Experiment 1, showing that perceptual averaging was predominantly biased by large, high-reward color singletons such that the absolute mean error was larger for this condition compared with conditions without a color singleton. Similarly, a difference in performance between high- and low-reward color singletons was observed, which could tentatively suggest that indeed adding an attention demanding task to the perceptual averaging task exposed the hypothesized differences between attentional allocation to high- and low-reward color singletons and its subsequent effect on perceptual averaging. Regardless of the precise nature underlying the differences in performance to high- and low-reward color singletons, these planned comparisons provide further, yet cautious support for the notion that reward can indeed differentially modulate perceptual averaging. However, the introduction of a secondary attention task did condemn the color singleton to a task-irrelevant distractor (for the search task, not for the averaging task) and in general, this appears to have reduced the overall effects of reward on perceptual averaging, such that only the most salient signals (high-reward color large circles) managed to influence this process. This somewhat weaker effect of reward on perceptual averaging is further exacerbated by the lack of a significant difference between the no-reward condition and either of the reward conditions (high or low).

One alternative explanation why the effects are not as straightforward as expected could be that the addition of a secondary task simply added more noise to the data. Indeed, the increased cognitive demands, such as increased working memory load (remembering the line orientation until response), or the need to refocus attention throughout the trial could potentially mask more pronounced effects that would allow for a more distinct differentiation between the role of physical salience and value-driven attentional effects on perceptual averaging. Importantly, these introduced factors are consistent throughout the experiment, and there is no reason to assume that the value-signaling components of the color singletons have changed. However, the manner in which the participant may act upon these stimuli in terms of attentional selection may differ.

## General discussion

Previous literature provides clear evidence that allocating attention to specific elements of a set modulates the set’s perceived mean sizes both in a top-down and bottom-up fashion (de Fockert & Marchant, 2008), and that such perceptual averaging can be influenced by value-signaling stimuli (Dodgson & Raymond, 2020). In the current study, selection history (as gauged with reward) has a similar effect on perceptual averaging, but the extent to which the average is biased is dependent on the ‘history’ of the attention capturing distractor. Both Experiment 1 and Experiment 2 show that large color singletons with the highest value (as a proxy of selection history) yield the largest biases in perceptual averaging. Low- and no-rewarding color singletons do not necessarily yield similar effects, despite their physical salience. The results of Experiment 1 clearly showed that the bias in the perceived average scales linearly with the increasing value of the attended color singleton. The introduction of a secondary, attention demanding task in Experiment 2, as a means to expose differences between the effects of high- and low-reward singletons on perceptual averaging, did not fully yield the expected results. However, results do suggest that preventing attention from being completely focused on task-irrelevant, reward signaling color singletons can indeed elicit the difference in the perceptual bias evoked by attention capturing stimuli with differing associated values. As such, the current work provides evidence that indeed selection history, like bottom-up and top-down attention, influences perceptual averaging, and that this happens in a flexible manner proportional to the extent to which attention is captured.

Note that the current results are fully driven by the large color singletons in both Experiment 1 and Experiment 2, whereas no effect was observed for the small color singletons. In retrospect, this is not unsurprising for several reasons. In the current experiment, the color singletons are equiluminant compared with the gray circles in the display, which leads to the color singleton being fully driven by differences in chromaticity, but not by color/luminance-based salience, which (together with abrupt onsets) are often deemed the features of a static stimulus responsible for attentional capture (Theeuwes, 1992, 2010). Furthermore, given that saliency is not a driving force behind capture in the current experiments, the minimal size of the small circles might have further reduced their attention capturing qualities, resulting in the absence of an effect of attention on the perceptual bias in this condition. To an extent, the lack of differences in physical salience between the color singletons and the gray circles in the display may also have contributed to the relatively weak effects observed for the large color singletons, as it has been hypothesized that value-driven capture may operate via an initial capture stage that is driven by physical salience, but where the moment of disengagement from the value-signaling distractor, and therefore the time attention lingers at the value-associated stimulus, is modulated by the stimulus–value association (Theeuwes & Belopolsky, 2012). While this explanation is far from implausible, alternative explanations need to be considered. For example, classic work by Bruner and colleagues (1947) has shown that poor children overestimate the perceived size of coins. That is, it appears that individual value-signaling stimuli are sometimes perceived as larger than they are. Overestimating the size of a value-signaling stimulus would increase the bias in the perceived mean size of a set when the value-signaling stimulus is larger than the mean of a set. However, when the value-signaling circle is smaller than the set mean, it may counter the value-based bias away from the mean, as the size overestimation of the small circle may influence the mean of the entire set. That is, the mean of the set is underestimated due to value-signaling small circle but overestimated due to perceiving value signaling stimuli as larger than they are. Such a claim is supported by recent work from Choi and Chong (2020), who showed that even if you make people attend to a single large/small item and that item causes under (small) or over (large) estimation relatively, the mean is still overall overestimated (referred to as perceptual enlargement). The net result of these two competing mechanisms may explain the lack of an effect for the small value-signaling circles used in the current experiment.

The current results are in line with the work by Dodgson and Raymond (2020) such that they provide further evidence that value-signaling stimuli tend to bias the perceived mean size of a set of stimuli. However, whereas the work by Dodgson and Raymond showed that the perceived average of a set of circles is biased when a subset of these circles is presented in a high-reward color, the current study shows that a single rewarded stimulus can yield similar biases. As such the current work makes perhaps a more convincing argument that the mechanism underlying value-induced biases of perceptual averages hinges on a selective attentional mechanism, where focusing on a single item within a set of circles biases the perception of the set towards the size of the attended circle. From a perceptual averaging point of view, such an explanation largely discards alternative explanations such as the use of subsampling strategies (Myczek & Simons, 2008) as the current design was specifically designed to avoid such a strategy due to presenting only one salient item (as opposed to Dodgson and Raymond). Nonetheless, despite focusing on an explanation suggesting that the current biases are the direct result of an attentional mechanism, this study does not rule out that other higher-order processing mechanisms do not also influence perceptual averaging. It could for example be argued that the special status of a value-signaling stimulus enhances its working memory representation such that it maintains access to conscious awareness, which in turn could alter the perception of a set’s statistical properties. Alternatively, perceptual averaging could be influenced by differentially weighting the various perceptual inputs as a result of lingering biases due to the initial reward-learning stages, with the perceptual processing of high-reward signaling stimuli being perceptually, not attentionally, prioritized over lower-reward signaling stimuli. Nonetheless, such differences between perception and attention may be subtle, and may even be supported by overlapping neural circuitry (e.g., Serences, 2008). However, based on the current research as well as previous work (de Fockert & Marchant, 2008) it is clear that attention can bias perceptual averaging, yet the precise relationship between these two mechanisms remains elusive.

While this and other work show that selective attention biases perceptual averaging, the relationship between attention and perceptual averaging appears to be more intricate. Perceptual averaging’s precise functional role within the larger scope of visual processing remains unclear. It is widely agreed that perceptual averaging allows the visual system a means for circumventing strict focused attention and processing capacity limitations. However, so far only one line of investigations has provided empirical evidence for a functional role of perceptual averaging in vision. Corbett and Melcher (2014) showed that perceptual averaging facilitated performance in a visual search task if a search display’s statistical properties stayed the same from one trial to the next, despite all individual elements changing from trial to trial (see also Corbett & Munneke, 2019). Repeating statistics over consecutive trials resulted in faster reaction times and fewer saccades when searching for a randomly located target. Interestingly, visual search tasks rely strongly on top-down attentional factors that facilitate the search process (when a target is not a color singleton pop-out). As such, a logical proposal would be that perceptual averaging and visual attention interact to optimize visual search. This hypothesis was tested by Corbett and Munneke (2019), using a paradigm similar to the one used by Corbett and Melcher (2014) with the crucial manipulation that while statistical properties such as mean size were held constant from trial-to-trial, the number of elements in the set was manipulated. The logic behind this manipulation was that if perceptual averaging and visual attention interact to facilitate visual processing then an increase in set size should lead to a less steep search slope for stable sets of trials as compared with unstable sets of trials. This would provide evidence that visual attention indeed has a cooperative relationship with perceptual averaging, working together to facilitate visual processing. However, no such results were observed. If anything, the work by Corbett and Munneke showed that attention and perceptual averaging have distinct, noninteracting influences on visual processing. Therefore, the notion that visual attention and perceptual averaging are influencing each other at a functional level of visual processing can be called into question. However, Corbett and Munneke’s study only tested the relationship between perceptual averaging and top-down attention in a difficult conjunction search task, which is far from an exhaustive overview of the attentional processing required for everyday vision and further research is needed to fully characterize the relationship between visual attention and perceptual averaging.

## Footnotes

1 As the order of the perceptual averaging task and the testing task varied over participants, we investigated whether there was an order effect on the observed error-scores of the perceptual averaging task. However, no such effects were observed (all *p*s > .05) and as such task order has not been further investigated.

2 While all statistical tests are generally conducted as two-tailed tests, where appropriate one-tailed tests are reported if such a test fits the underlying directional hypothesis.

3 As the order of the perceptual averaging task and the testing task varied over participants, we investigated whether there was an order effect on the observed error scores of the perceptual averaging task. However, no such effects were observed (all *p*s > .05) and as such task order has not been further investigated.
